# Glycocalyx sialic acids regulate Nrf2-mediated signaling by fluid shear stress in human endothelial cells

**DOI:** 10.1016/j.redox.2020.101816

**Published:** 2020-11-28

**Authors:** Paraskevi-Maria Psefteli, Phoebe Kitscha, Gema Vizcay, Roland Fleck, Sarah J. Chapple, Giovanni E. Mann, Mark Fowler, Richard C. Siow

**Affiliations:** a King's British Heart Foundation Centre of Research Excellence, School of Cardiovascular Medicine & Sciences, Faculty of Life Sciences & Medicine, King's College London, London, SE1 9NH, United Kingdom; b Centre for Ultrastructural Imaging, Faculty of Life Sciences & Medicine, King's College London, London, SE1 1UL, United Kingdom; c Strategic Science Group, Unilever R&D, Colworth Science Park, Bedford, MK44 1LQ, United Kingdom

**Keywords:** Endothelial glycocalyx, Glutamate-cysteine ligase, Heme oxygenase-1, Hemodynamic shear stress, Mechanotransduction, NAD(P)H quinone oxidoreductase-1, Neuraminidase, Nrf2, Sialic acid

## Abstract

Activation of the nuclear factor erythroid 2–related factor 2 (Nrf2) pathway is critical for vascular endothelial redox homeostasis in regions of high, unidirectional shear stress (USS), however the underlying mechanosensitive mediators are not fully understood. The endothelial glycocalyx is disrupted in arterial areas exposed to disturbed blood flow that also exhibit enhanced oxidative stress leading to atherogenesis. We investigated the contribution of glycocalyx sialic acids (SIA) to Nrf2 signaling in human endothelial cells (EC) exposed to atheroprotective USS or atherogenic low oscillatory shear stress (OSS). Cells exposed to USS exhibited a thicker glycocalyx and enhanced turnover of SIA which was reduced in cells cultured under OSS. Physiological USS, but not disturbed OSS, enhanced Nrf2-mediated expression of antioxidant enzymes, which was attenuated following SIA cleavage with exogenous neuraminidase. SIA removal disrupted kinase signaling involved in the nuclear accumulation of Nrf2 elicited by USS and promoted mitochondrial reactive oxygen species accumulation. Notably, knockdown of the endogenous sialidase NEU1 potentiated Nrf2 target gene expression, directly implicating SIA in regulation of Nrf2 signaling by USS. In the absence of SIA, deficits in Nrf2 responses to physiological flow were also associated with a pro-inflammatory EC phenotype. This study demonstrates that the glycocalyx modulates endothelial redox state in response to shear stress and provides the first evidence of an atheroprotective synergism between SIA and Nrf2 antioxidant signaling. The endothelial glycocalyx therefore represents a potential therapeutic target against EC dysfunction in cardiovascular disease and redox dyshomeostasis in ageing.

## Highlights

• Oscillatory but not laminar shear stress reduces endothelial glycocalyx sialic acid.
• Laminar shear stress activates Nrf2-regulated endogenous antioxidant defences.
• Disruption of sialic acids attenuates Nrf2 activation by laminar shear stress.
• Knockdown of endogenous sialidase NEU1 enhances Nrf2 responses to flow.
• The glycocalyx maintains endothelial redox homeostasis in response to shear stress.

## Graphical abstract

**Figure:**
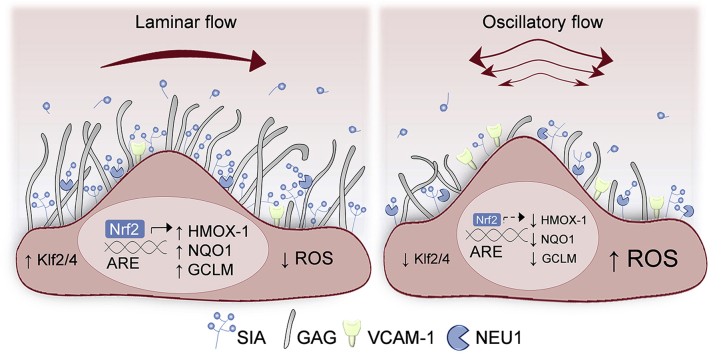

## Abbreviations

ARE: antioxidant response element
EC: endothelial cell
GAG: glycosaminoclycan
GCLM: glutamate-cysteine ligase modifier subunit
GCX: glycocalyx; HO-1, heme oxygenase-1
HS: heparan sulfate
KEAP1: Kelch-like ECH-associated protein 1
Klf: Krüppel-like factor
NEU1: Neuraminidase-1
NF-κB: Nuclear transcription factor-κB
NQO-1: NAD(P)H quinone oxidoreductase-1
Nrf2: Nuclear factor erythroid 2–related factor 2
OSS: oscillatory shear stress
ROS: reactive oxygen species
SIA: sialic acid
USS: unidirectional shear stress
VCAM-1: Vascular cell adhesion molecule-1

## 1 Introduction

Vascular endothelial cell defences against oxidative stress are coordinated by the transcription factor Nrf2, which modulates antioxidant gene expression through binding to DNA sequences termed antioxidant response elements (ARE) [1]. Under basal conditions, proteasomal degradation of constitutively synthesised Nrf2 is mediated by its cytosolic redox-sensitive partner Kelch-like ECH-associated protein 1 (Keap-1) [2]. Cytotoxic insults such as electrophiles and xenobiotics disrupt this interaction [3,4], allowing Nrf2 to accumulate in the nucleus where it promotes the transcription of genes encoding antioxidant, phase II detoxifying and glutathione synthesising enzymes to restore redox balance [5].

As reviewed previously [6], endothelial Nrf2 signaling is promoted by high unidirectional shear stress (USS) [7], whereas arterial regions exposed to low oscillatory shear stress (OSS) are prone to atherogenesis, partly due to diminished endothelial nitric oxide synthase (eNOS) expression [8] and attenuated antioxidant and anti-inflammatory properties of Nrf2 activation [9]. Exposure of EC to USS has been shown to promote Nrf2-dependent induction of cytoprotective genes [10], due to oxidation of thiol groups on Keap-1 [11] by cellular sources of reactive oxygen species (ROS) [12,13]. Activation of the Nrf2 pathway by USS can also be mediated by kinase signaling events [14,15] and is primed by shear-sensitive expression of Krüppel-like factor 2 (Klf2) [16], responsible for transcriptional programing of endothelial atheroprotection [17]. In contrast, Nrf2 stabilization and nuclear translocation in response to OSS does not promote ARE-dependent gene transcription [12] due to additional epigenetic regulation by histone deacetylases and mechano-sensitive microRNAs [6].

Despite the pivotal role of shear-sensitive Nrf2 regulation in determining susceptibility to vascular disease, the biomechanical mediators of this effect remain to be fully elucidated. Various plasma membrane molecules, microdomains and cytoskeletal components participate in shear stress mechano-sensation and transduction [18]. In particular, the glycocalyx (GCX), comprised of glycoproteins, proteoglycans, glycosaminoglycans (GAG) and glycolipids, has dimensions and biochemical composition that dependent on the dynamic equilibrium between its biosynthesis, degradation and local shear stress profiles [19]. The GCX contributes to the regulation of vascular tone via its mechanotransduction properties and is critical for blood rheology in the microcirculation, molecular filtration across the vascular wall, as well as thromboresistance and immuno-modulation [20]. Sialic acid (SIA) monosaccharides occupy the terminal branches of glycan chains within the GCX of EC, blood cells and common pathogens [21]. Arterial segments exposed to disturbed shear stress exhibit SIA deterioration, which predisposes them to atherogenesis [22]. Diminished SIA in the endothelial GCX is also associated with an enhanced risk of vascular dysfunction in diabetes [23] and is observed in rodent models of ageing [24]. Notably, EC desialylation by exogenous sialidases has been shown to impair NO-dependent vasodilatation by shear stress [25] due to enhanced ROS generation [26]; however the contribution of SIA in shear mediated induction of endogenous antioxidant defences remains to be elucidated.

In this study, we report the first evidence that fluid shear stress regulates EC redox signaling via alterations in the SIA component of the GCX. Using primary human EC, we demonstrate differential SIA expression and Nrf2-mediated antioxidant responses to USS and OSS. Furthermore, cleavage of SIA by exogenous neuraminidase led to diminished USS-mediated Nrf2 activation and an enhanced pro-inflammatory EC phenotype. In contrast, silencing of endogenous sialidase NEU1 enhanced Nrf2 responses to flow, highlighting the shear-sensitive crosstalk between SIA and endogenous antioxidant defences. Our findings demonstrate that OSS-mediated SIA modifications lead to diminished activation of atheroprotective Nrf2 signaling, suggesting that GCX could be a key therapeutic target not only for age-related cardiovascular disease (CVD) but also infectious diseases, cancer and diabetes.

## 2 Materials and methods

### 2.1 Materials and reagents

Neuraminidase from *Clostridium perfringens (C. welchii)* and β-actin antibody were obtained from Millipore-Sigma (Burlington, MA, USA). CF™488A-WGA and CF™568A-PNA lectins were from Biotium Inc. (Hayward, CA, USA), heparan sulfate (HS) epitope 10E4 antibody was from AMS Biotechnology (Abingdon, UK) and HO-1 antibody from BD Biosciences (San Jose, CA, USA). Nrf2, eNOS, NQO1, Klf2 and Klf4 antibodies as well as polybrene and puromycin were obtained from Santa Cruz Biotechnology Inc. (Dallas, TX, USA). Phospho-Nrf2 (S40), VCAM-1, NFkB (p65) and all AlexaFluor® secondary antibodies were from Abcam (Cambridge, UK). GCLM antibody was a kind gift of Prof. Terrance Kavanagh (University of Washington, Seattle). Phospho-GSK3β (Y216) and total GSK3β, phospho-eNOS (S1177 and S633), phospho-protein kinase B (Akt, S473) and total Akt antibodies were from Cell Signaling Technology (Danvers, MA, USA). Enhanced chemiluminescence reagents (ECL) were from GE Healthcare Life Science (Amersham, UK). All other chemicals, reagents and tissue culture supplies were purchased from Millipore-Sigma (Burlington, MA, USA).

### 2.2 Endothelial cell isolation and culture

Umbilical cords from healthy, full-term pregnancies were obtained from the Maternity Unit at St. Thomas’ Hospital (London, UK) with informed participant consent and Research Ethics Committee approval (Ref:15/EM/0290). Human umbilical vein endothelial cells (HUVEC) were isolated within 2 days of delivery using collagenase digestion as previously described [27]. Cells were cultured in gelatin-coated flasks in Medium 199 (M199) containing 10% (v/v) fetal and 10% (v/v) neonatal calf serum, NaHCO_3_ (18 mmol L^-1^), penicillin/streptomycin (119 U ml^-1^/120 μg ml^-1^), l-glutamine (5 mmol L^-1^) and endothelial cell growth supplement (ECGS, 5 ng ml^-1^) in a 5% CO_2_/95% humidified air incubator at 37 °C. EC monolayers were passaged with trypsin and all experiments were performed at passage 3. The HUVEC-derived endothelial cell line EA. hy926 (gifted by Unilever UK) [28] was used for infection with lentiviral vectors and maintained under the same conditions as HUVEC.

### 2.3 Fluid shear stress application

The Ibidi parallel-plate flow system (Ibidi GmbH, Germany) was used to recapitulate the laminar and oscillatory shear stress profiles associated with anti- and pro-atherogenic EC phenotypes, respectively. As the endothelial glycocalyx is established upon reaching quiescence [29], EC seeded in μ-I^0.6^ Luer slides were maintained in static culture for 48 h to allow sufficient GCX growth. Cell monolayers were then exposed to shear stress (τ, dynes cm^-2^), calculated using the formula τ = μ 60.1 Φ. τ is proportional to the dynamic viscosity of the medium μ (0.00782 dyn s cm^-2^ for M199 at 37 °C [30]) and the flow rate Φ (ml min^-1^) generated by the air pressure pump. Cells were preconditioned to two consecutive 30 min cycles of 2 and 5 dyn cm^-2^ of unidirectional flow (USS), followed by either disturbed flow of ±5 dyn cm^-2^ where the direction reverses periodically (OSS, 1Hz oscillations) or USS of 15 dyn cm^-2^, each for the indicated experimental periods. All cells were incubated in M199 without ECGS (basal M199) for 12 h before and during shear stress application.

### 2.4 Transmission electron microscopy (TEM)

Changes in GCX size and organisation were assessed by TEM at the Centre for Ultrastructural Imaging (King's College London). To preserve GCX integrity HUVEC were cultured on the detachable bottom of an Ibidi sticky slide μ-I^0.6^ Luer and were perfused with two lysine-acetate solutions; the first containing 2% glutaraldehyde and 0.08% Alcian Blue (AB), followed by one containing 2% paraformaldehyde, 2.5% glutaraldehyde and 0.075% Ruthenium Red (RR). AB, RR and lysine are cationic reagents with high affinity for the negatively charged GCX [31]. Samples were osmicated (1% OsO_4_) and dehydrated in a graded ethanol series before embedding in epoxy resin. Ultrathin (70–90 nm) sagittal sections obtained with a Leica UC7 ultramicrotome were mounted on 150 μm mesh copper grids and double-contrasted in UranyLess and 3% lead citrate (Electron Microscopy Sciences, UK) before examination under a JEM-1400Plus microscope (JEOL). High power electron micrographs of at least 10 different cells per condition were analysed using FIJI software [32]. Twenty measurements were collected from each cell, at points where both phospholipid bilayers were visible to ensure that luminal GCX depth was measured perpendicular to the plasma membrane [33]. GCX thickness was determined as half the maximal pixel intensity of the distance between the luminal edge and the lipid bilayer. Occasionally strands extending up to 200 nm were visible that were not included in the quantification process.

### 2.5 SIA cleavage and staining

SIA removal was achieved enzymatically using neuraminidase from *C. perfringens* as described previously [34]. EC were incubated in basal M199 lacking all serum that contained 2U ml^-1^ neuraminidase (Sigma units) for 30 min at 37 °C. Cells were then either returned to basal M199 for further experimentation or fixed with 4% paraformaldehyde (PFA) for 10 min. Subsequently, cells were blocked with 4% bovine serum albumin (BSA) at room temperature (RT, 1 h) and stained with wheat germ agglutinin (WGA) conjugated to a green fluorescent dye (CF™488A, excitation/emission: 490/515 nm, 2 μg ml^-1^, 30 min). Cell nuclei were stained with 4, 6-diamidino-2-phenylindole dihydro chloride (DAPI, 2 μg ml^-1^) and samples were preserved in mounting media (Ibidi GmbH, Germany) until imaged.

WGA images (2048 × 2048 pixels) were acquired with LSM-780 confocal laser scanning microscope (AxioObserver.z1, Carl Zeiss GmbH, Germany) using an oil immersion objective (Zeiss, Plan-Apochromat ×40/1.3 NA). WGA-CF^TM^448A and DAPI were excited with Argon (458/488/514 nm, 25 mW) and diode (405 nm, 30 mW) lasers, respectively. For some experiments, planar sections were obtained along the z-axis (0.1 μm apart) and reconstructed into orthogonal views with FIJI software which was also used for false coloring and image analysis. For some experiments, SIA and DAPI were visualized with an epifluorescence microscope as described below. The mean fluorescence intensity of background subtracted fields of view (FOV) was normalized to the respective number of cell nuclei and expressed as mean cell intensity (MCI) of at least 300 cells per experimental condition.

### 2.6 Determination of free SIA

The total amount of free SIA was determined enzymatically using the NANA Assay kit (Abcam, Cambridge, UK) according to the manufacturer's protocol. Briefly, conditioned cell culture medium was centrifuged (1500 rpm, 5 min) and equal volumes of sample or assay standard were allowed to react (RT, 30 min) with the Oxi-Red probe that relies on free SIA oxidation to give fluorescence at excitation/emission:535/587 nm that was measured with a plate reader (ClarioStar; BMG Labtech, Germany). The concentration of free SIA was normalized to the original sample volume.

### 2.7 RT-qPCR

The *mir*Vana™ miRNA isolation kit (Ambion, Thermo Fisher Scientific) was used to extract total RNA which was reverse transcribed in equal amounts (300 ng) with the high capacity cDNA kit (Applied Biosystems™, Thermo Fisher Scientific). Gene expression was determined with SYBR® green I (Sensimix™ No-ROX kit, Bioline) using specific primer pairs (Table 1) and amplified by Rotor-Gene™ 6000 thermal cycler (Corbett Research, UK). Target gene levels were interpolated by a standard curve of known copy number concentrations and normalized to the geometric mean of five reference genes using geNorm algorithm [35] (Table 1).

**Table 1:** List of primer sequences.

Gene	Forward	Reverse
KLF2	5′-CGCTGAGTGAACCCATCCTG-3′	5′-ATGAAGTCCAGCACGCTGTT-3′
KLF4	5′-GCCGCTCCATTACCAAGAG-3′	5′-GTAATCACAAGTGTGGGTGGC-3′
GNE	5′-CACAGGCACAGGAATCGGT-3′	5′-CCATTCCAGAGGCGTATGCT-3′
CMAS	5′-TCGTGAAGTGACCGAACCTC-3′	5′-TTCCACCCTGCAAGTAACCC-3′
SLC35A1	5′-TTGTGACATTAGCTGGCGTCT-3′	5′-GCAAGAAAGATGACAAACCAGACA-3′
NEU1	5′-GCACATCCAGAGTTCCGAGT-3′	5′-CAGGGTTGCCAGGGATGAAT-3′
**Reference genes**		
ACTB	5′-CCAGAGGCGGTACAGGGAATAG-3′	5′-CCAACCGCGAGAAGATGA-3′
B2M	5′-TTCTGGCCTGGAGGCTATC-3′	5′-TCAGGAAATTTGACTTTCCATTC-3′
RPL13A	5′-GAGGCCCCTACCACTTCC-3′	5′-AACACCTTGAGACGGTCCAG-3′
SDHA	5′-AGAAGCCCTTTGAGGAGCA-3′	5′-CGATTACGGGTCTATATTCCAGA-3′
TBP	5′-GCTGGCCCATACTGATCTTT-3′	5′-CTTCACACGCCAAGAAACAGT-3′

***Abbreviations:*** ACTB, β-actin; B2M, β-2-microglobulin; CMAS, cytidine monophosphate N-acetylneuraminic acid synthetase; GNE, glucosamine (UDP-N-acetyl)-2-epimerase/N-acetylmannosamine kinase; KLF2/4, Krüppel-like factor 2/4; NEU1, neuraminidase 1; RPL13A, 60S ribosomal protein L13A; SDHA, succinate dehydrogenase complex subunit A; SLC35A1, solute carrier family 35 member A1; TBP, TATA-box binding protein.

### 2.8 Immunoblotting

Whole-cell protein was extracted with a sodium dodecyl sulfate lysis buffer (2% w/v) containing protease and phosphatase inhibitors. Total protein content was determined with the bicinchoninic acid assay (Pierce, Thermo Fisher Scientific) before separation of equal amounts of denatured protein by gel electrophoresis and transfer to PVDF membranes. Non-specific binding sites were blocked with 5% w/v skimmed milk before overnight incubation (4 °C) with primary antibodies raised against proteins of interest or β-actin that was used as a loading control. Protein expression was detected with horseradish peroxidase-conjugated secondary antibodies and countered by ECL reagents. Immunoblots were visualized with the G-box gel documentation system (Syngene Bioimaging) and band densitometric analysis was carried out with FIJI software.

### 2.9 Lentiviral gene transfection

EA.hy926 cells were infected at multiplicity of infection of 10 (48 h) with lentiviral particles containing anti-Nrf2, anti-NEU1 or non-target (scrambled) shRNA and puromycin resistance genes (Santa Cruz Biotechnology). Transfection efficiency was enhanced with of polybrene (5 μg ml^-1^) and following 72-h recovery in antibiotic-free M199, stably transduced cells were identified by puromycin (2 μg ml^-1^) selection. Antibiotic-resistant cell populations were expanded over two weeks in the continuous presence of puromycin, which was removed 48 h before experimentation.

### 2.10 Nrf2 and NFkB immunocytochemistry

Fixed cells were permeabilized with TritonX-100 (0.1%, 10 min) and blocked with 4% BSA. Cellular localization of Nrf2 or the p65 (Rel-A) subunit of NFkB was examined by specific primary (overnight, 4 °C) and AlexaFluor® 488 and 555 secondary antibodies (1 h at RT), respectively. Immunofluorescence images were acquired with a water immersion objective (Olympus, LUMPlanFL ×40/0.8 NA) of an inverted epifluorescence microscope (Nikon Diaphot) fitted with a Nikon DXM1200F digital camera. Nuclear and cytoplasmic fluorescence intensity were quantified and background corrected using FIJI software.

### 2.11 Detection of mitochondrial ROS

At the end of each experimental protocol, cells were loaded with the dihydroethidium-conjugated fluorogenic probe MitoSOX^TM^Red (excitation/emission: 510/580 nm, Invitrogen) prepared in serum-free M199 (5 μΜ). Incubation for 30 min under static conditions was followed by fixation with 4% PFA and nuclei staining with DAPI before imaging with an inverted epifluorescence microscope as described above. FIJI software was used to quantify and background correct the mean fluorescence intensity that was normalized to the number of cell nuclei per FOV.

### 2.12 Statistics

Data denote mean ± S.E.M. from experiments of at least 3 different HUVEC donors or 3 independent EA. hy926 cultures, unless otherwise stated. Statistical comparisons between two independent groups were performed with unpaired Student's *t*-test while one- or two-way ANOVA with Tukey or Bonferroni post hoc tests were used to evaluate statistical differences between more than two conditions. P values < 0.05 were considered statistically significant.

## 3 Results

### 3.1 Laminar flow enhances luminal GCX expression *in vitro*

**Fig. 1:**
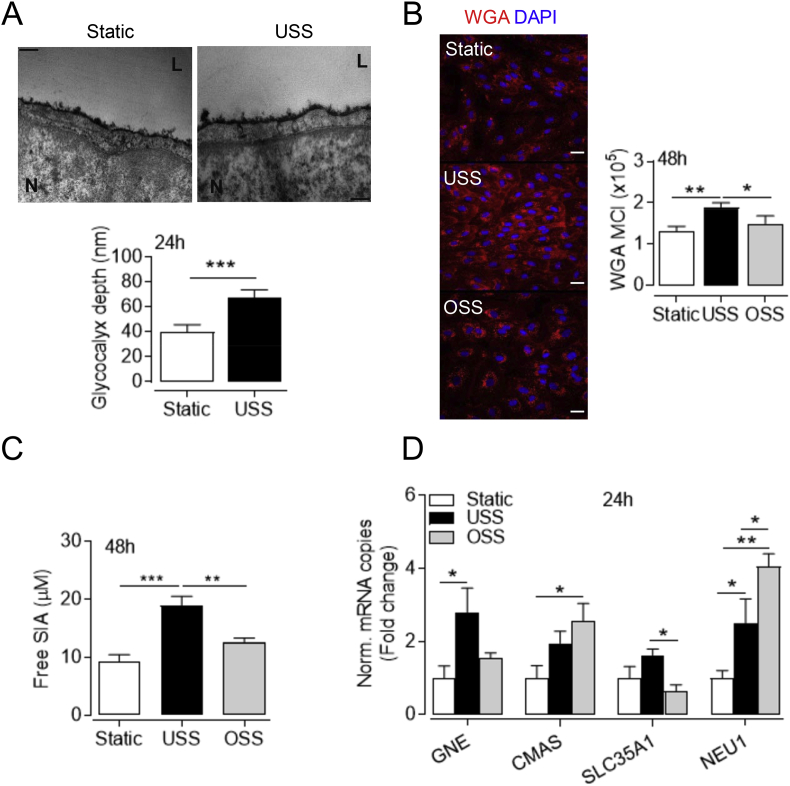
Differential effects of laminar and disturbed shear stress on SIA expression. HUVEC were exposed to USS (15 dyn cm^-2^), OSS (±5 dyn cm^-2^, 1 Hz) or maintained in static conditions for 24 or 48 h, as indicated. (**A**) Representative electron micrographs of the cross-sectional aspect of the luminal GCX stained with ruthenium red and Alcian blue. Twenty measurements of the luminal GCX depth were averaged from each cell and are expressed as mean ± S.D. (n = 1 donor) from at least 10 different cells per condition. ***P < 0.001 (Student's *t*-test). L, channel lumen; N, nucleus. *Scale bar=*200 nm. (**B**) Representative confocal images of the SIA component of the GCX stained with WGA-CF^TM^448A (red) in fixed cells. WGA mean cell intensity (MCI) was quantified in the x-y optical slices and normalized to the respective number of cell nuclei stained with DAPI (blue). Data represent mean ± S.E.M. (n = 5 different donors). *P < 0.05; **P < 0.01 (1-way ANOVA). *Scale bar=*20 μm. (**C**) Amount of free SIA in the conditioned culture media was assessed fluorometrically. Data are mean ± S.E.M. (n = 6 different donors). **P < 0.01; ***P < 0.001 (1-way ANOVA). (**D**) Relative mRNA expression of the genes GNE, CMAS, SLC35A1 and NEU1 encoding enzymes involved in SIA biosynthesis, transport and cleavage was determined by real time-PCR and normalized to 5 reference genes. Data are expressed as fold change from respective mRNA levels in static culture and denote mean ± S.E.M. (n = 4–5 different donors). *P < 0.05; **P < 0.01 (1-way ANOVA). (For interpretation of the references to color in this figure legend, the reader is referred to the Web version of this article.)

Although HUVEC maintained in culture have diminished GCX thickness compared to the umbilical vein *in vivo* [36], multiple studies have demonstrated the vasoprotective properties of the GCX *in vitro*. Since static culture does not represent the dynamic conditions developed in the vasculature, we exposed HUVEC to laminar flow to more accurately recapitulate the physiological GCX environment. As revealed by TEM, HUVEC maintained in static culture have a rudimentary GCX, which becomes more uniform and thicker after prolonged exposure to USS (Fig. 1A). SIA is a major component of the vascular GCX and is markedly reduced in atheroprone regions of the vasculature exposed to disturbed shear stress profiles [37]. Given the abundance of SIA in the human umbilical vein [38], we next assessed its expression using WGA lectin labelling. Consistent with previous studies, static cells had abundant SIA expression at different passages (p0 to p3, data not shown) and while application of USS enhanced SIA levels (Fig. 1B), WGA intensity was diminished in cells exposed to OSS. In line with the TEM findings above, the 3-dimensional volume views of the confocal reconstructions, demonstrated enhanced apical localization of WGA staining in response to laminar flow (Fig. S1), however, that was not observed under static or OSS conditions.

To ascertain whether shear stress-modulation of WGA binding is due to altered SIA expression and not a consequence of stereochemical changes affecting their interaction [39], the free SIA content of the conditioned culture medium was investigated. Free SIA was accumulated in the culture medium of cells exposed to USS relative to OSS or static culture (Fig. 1C). Because glycan sialylation depends on the balance between SIA biosynthesis and the opposing actions of endogenous sialyltransferases and sialidases, the transcription of genes encoding enzymes involved in SIA metabolism was also assessed (Fig. 1D). mRNA levels of the rate-limiting SIA biosynthetic enzyme glucosamine (UDP-N-acetyl)-2-epimerase/N-acetylmannosamine kinase 1 (GNE) [40] paralleled the differential SIA expression in response to USS and OSS. In contrast, only OSS enhanced mRNA expression of the nuclear enzyme cytidine monophosphate N-acetylneuraminic acid synthetase (CMAS) which generates SIA and nucleotide sugar pairs [41]. These are subsequently transported into the Golgi via the SLC35A1 antiporter [42] that was here reduced at mRNA level in response to OSS. Consistent with previous reports [43], the endogenous sialidase NEU1, which hydrolyses terminal SIA from the adjacent glycans, was the most abundant isoform expressed in HUVEC (data not shown). NEU1 mRNA was enhanced under flow conditions (Fig. 1D), but to a significantly greater extent by disturbed rather than laminar flow. These observations highlight that SIA remodelling is particularly susceptible to variations in shear stress and thus may regulate mechano-sensitive signaling.

### 3.2 OSS and SIA disruption attenuate antioxidant Nrf2 signaling activation by physiological flow

**Fig. 2:**
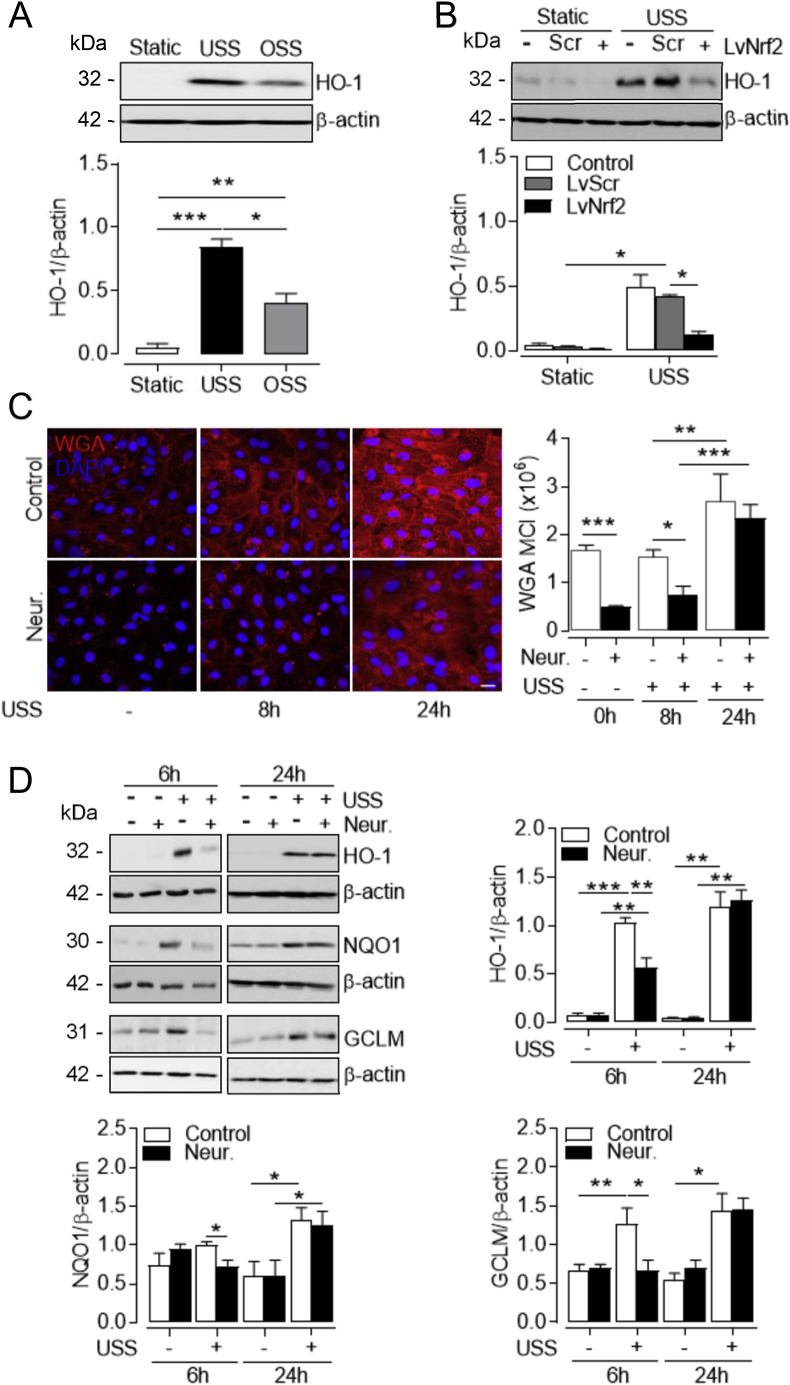
Induction of Nrf2 signaling by USS depends on SIA integrity. (**A**) Representative immunoblot of HO-1 expression in HUVEC subjected to USS (15 dyn cm^-2^), OSS (±5 dyn cm^-2^, 1 Hz) or maintained in static culture for 48 h. Densitometric analysis of HO-1 levels is shown relative to β-actin. Data are mean ± S.E.M. (n = 3 different donors). *P < 0.05; **P < 0.01; ***P < 0.001 (1-way ANOVA). (**B**) EA. hy926 cells stably transduced with lentiviral particles containing Nrf2 silencing shRNA (LvNrf2), scrambled sequences (Scr) or left untransfected (Control) were exposed to USS (15 dyn cm^-2^) for 24 h. HO-1 expression was assessed by immunoblotting relative to β-actin. Data denote mean ± S.E.M. (n = 3 independent experiments). *P < 0.05 (2-way ANOVA). (**C–D**) Following SIA removal with neuraminidase (Neur., 2 U ml^-1^, 30 min), both control and Neur.-treated HUVEC cultures were exposed to USS (15 dyn cm^-2^) for 6, 8 and 24 h or immediately terminated (0 h), as indicated. (**C**) Representative confocal images of SIA stained with WGA-CF^TM^448A (red) in fixed HUVEC. WGA mean cell intensity (MCI) was quantified in the x-y optical slices and normalized to the number of cell nuclei stained with DAPI (blue). Data denote mean ± S.E.M. (n = 3–6 different donors). *P < 0.05; **P < 0.01; ***P < 0.001 (2-way ANOVA). *Scale bar=*20 μm. (**D**). Protein expression and densitometric analyses of the Nrf2 targets HO-1, NQO1 and GCLM relative to β-actin. Data denote mean ± S.E.M. (n = 4–5 different donors). *P < 0.05; **P < 0.01; ***P < 0.001 (2-way ANOVA). (For interpretation of the references to color in this figure legend, the reader is referred to the Web version of this article.)

One of the most characterized Nrf2 targets is heme oxygenase-1 (HO-1), a stress response enzyme transcriptionally upregulated to confer protection against oxidative damage [5]. Physiological shear stress is a potent inducer of HO-1 expression, which is diminished in vascular areas susceptible to atherosclerosis and cells exposed to disturbed flow [7,9]. In line with these studies, HO-1 was upregulated in HUVEC exposed to flow, but the response to physiological USS was significantly greater (Fig. 2A). Under these conditions, similar results were obtained for the Nrf2-regulated detoxifying enzyme NAD(P)H quinone 1 oxidoreductase 1 (NQO1) and the modifier subunit of glutamate cysteine ligase (GCLM) required for biosynthesis of glutathione (Fig. S2A). Flow-mediated changes in antioxidant enzyme expression were also associated with increased Nrf2 nuclear localization in response to USS compared to OSS and static culture (Fig. S2B). To confirm that upregulation of these antioxidant enzymes is Nrf2 dependent, lentiviral silencing of Nrf2 was achieved in the shear-responsive EA. hy926 endothelial cell line which abolished USS upregulation of total Nrf2 protein levels (Fig. S2C). USS-mediated induction of HO-1 protein expression was also abrogated in Nrf2 knockdown cells (Fig. 2B), and this was replicated for NQO1 and GCLM expression (Fig. S2C) and with shorter exposure to USS (data not shown).

Our study established a similar temporal regulation of the Nrf2/ARE pathway and SIA expression within the same 48-h period of shear stress conditioning. As enzymatic removal of SIA enhances ROS production [26], we next investigated whether this is due to mechanosensitive modulation of the Nrf2/ARE pathway. To selectively remove the SIA component of the GCX, cells were treated with 2U ml^-1^ neuraminidase (Fig. 2C and Fig. S3) for a short period of time (30 min) to avoid the observed time- and dose-dependent decline in cell viability (data not shown). As GCX regrowth is a dynamic process with distinct recovery timeline for different components [44] and is enhanced in response to physiological flow [45], control and neuraminidase-treated cultures were subjected to USS for different times. Cell exposure to USS enhanced WGA staining at 24 h compared to 8 h in both treatment conditions (Fig. 2C). Neuraminidase significantly decreased WGA staining for at least 8 h post treatment, but significant SIA restoration was observed after 24 h (Fig. 2C). To avoid cytotoxicity caused by continuous exposure to neuraminidase, we used the acute SIA recovery period (<8 h) to assess its role in Nrf2 signaling. SIA removal with neuraminidase attenuated the induction of HO-1, NQO1 and GCLM protein levels in response to 6 h of USS (Fig. 2D). After 24 h, however, the induction of these enzymes by laminar shear stress was restored, concomitant with regrowth of SIA. Notably, treatment with neuraminidase under static conditions did not affect antioxidant enzyme expression at similar time points assessed (data not shown).

### 3.3 SIA removal impairs Nrf2 nuclear accumulation in response to physiological flow

**Fig. 3:**
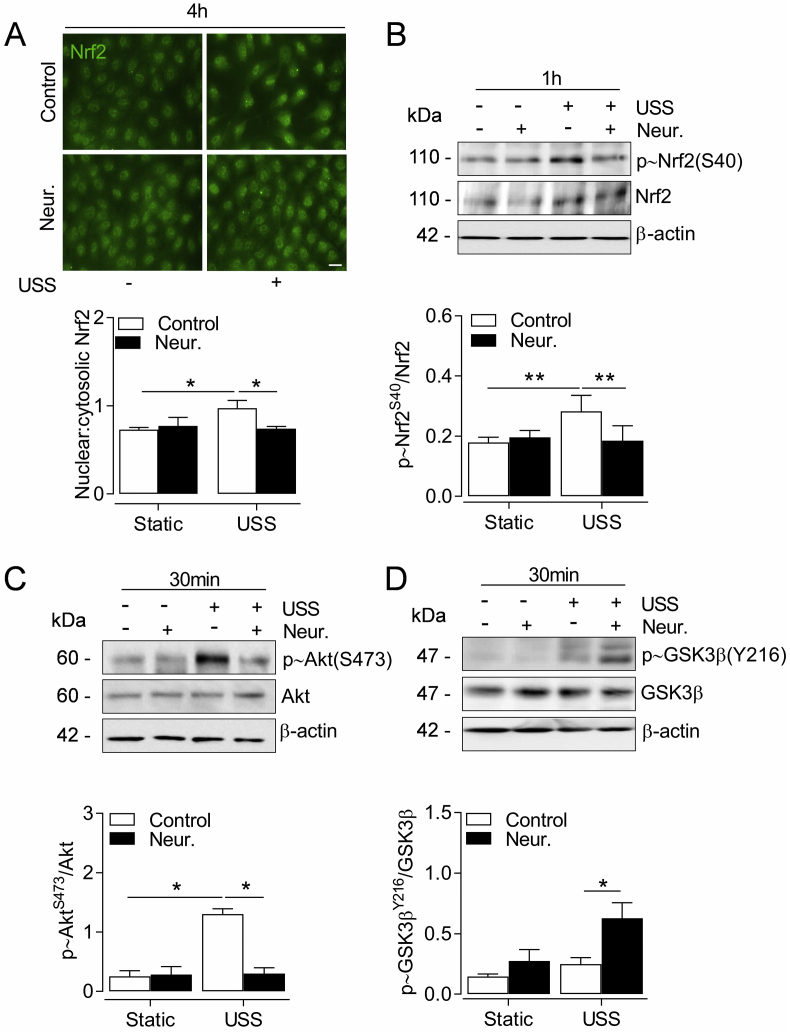
Effect of SIA cleavage on Nrf2 nuclear translocation and kinase activation by USS. HUVEC were incubated with neuraminidase (Neur., 2 U ml^-1^, 30 min) before exposure to USS (15 dyn cm^-2^) for the indicated time periods. (**A**) Representative images of Nrf2 cellular localization and nuclear-to-cytosolic fluorescence intensity ratio. Cell nuclei were stained with DAPI which was omitted for clarity. Data are mean ± S.E.M. (n = 5 donors) of quantification analysis from at least 300 cells per condition. *P < 0.05 (2-way ANOVA). *Scale bar=*20 μm. (**B-D**) Representative immunoblots and densitometric analyses of phosphorylation of Nrf2-S40 (**B**), Akt-S473 (**C**) and GSK3β-Y216 (**D**) relative to respective total protein and β-actin loading controls. Data denote mean ± S.E.M. (n = 4–6 different donors). *P < 0.05; **P < 0.01 (2-way ANOVA).

We next investigated the mechanisms by which SIA regulate Nrf2 signaling. USS stabilises newly synthesised Nrf2 protein and enhances its nuclear accumulation [10,13], which we observed after 4 h of USS application (Fig. 3A). SIA removal with neuraminidase significantly reduced nuclear Nrf2 levels in cells exposed to USS (Fig. 3A). Moreover, neuraminidase attenuated USS-induced phosphorylation of Nrf2-Ser40 (Fig. 3B), previously shown to reduce Nrf2 association with Keap-1 in cells maintained in static culture [46]. In response to physiological flow the latter mechanism is mediated by protein kinase B (Akt) activity [7]. Indeed, acute exposure of HUVEC to USS stimulated Akt phosphorylation, which was significantly reduced following SIA cleavage with neuraminidase (Fig. 3C). Activation of the phosphoinositide 3-kinase (PI3K)–Akt pathway can further promote Nrf2 activity via inhibition of glycogen synthase kinase 3β (GSK3β) [47]. When HUVEC were treated with neuraminidase prior to USS exposure, phosphorylation of GSK3β at Tyr216 was increased (Fig. 3D).

### 3.4 NEU1 knockdown promotes Nrf2 activation by physiological flow

**Fig. 4:**
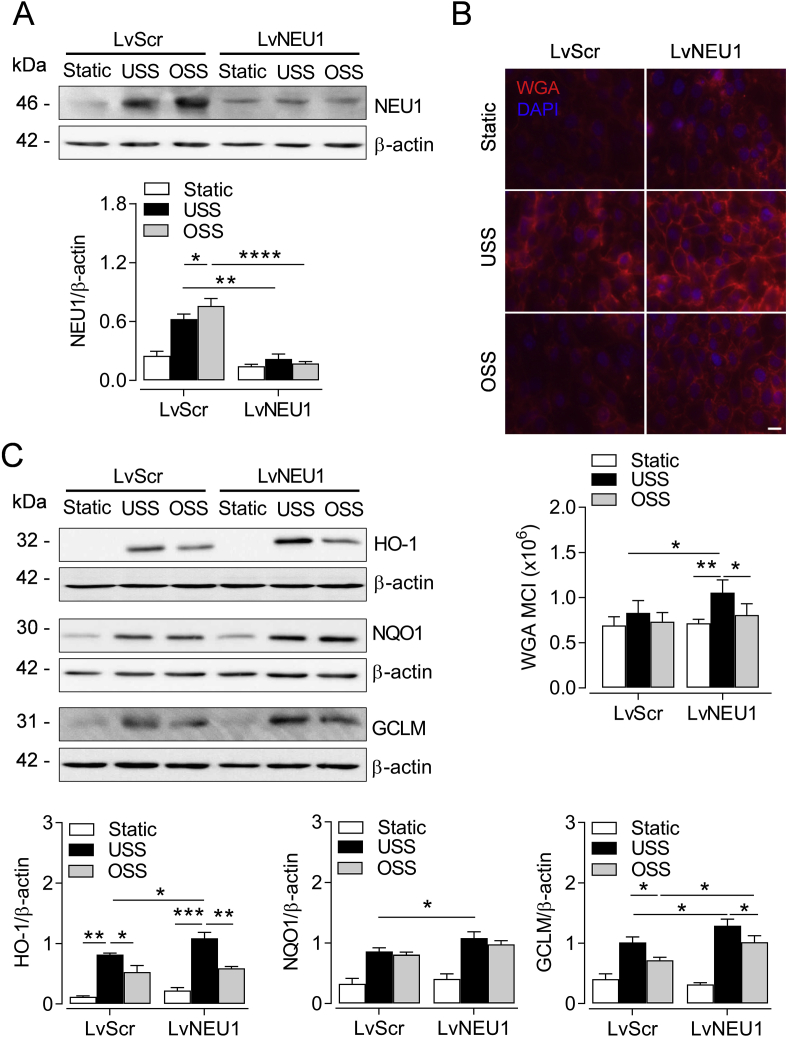
Endogenous NEU1 knockdown enhances Nrf2-mediated antioxidant signaling. EA. hy926 cells transduced with lentiviral particles either containing sialidase 1 (NEU1) silencing shRNA (LvNEU1) or scrambled sequences (Scr) were exposed to USS (15 dyn cm^-2^), OSS (±5 dyn cm^-2^, 1 Hz) or maintained in static culture for 48 h. (**A**) Representative immunoblot and densitometric analysis of NEU1 expression in whole cell lysates presented relative to β-actin. Data denote mean ± S.E.M. (n = 5 independent cultures). *P < 0.05; **P < 0.01; ***P < 0.001 (2-way ANOVA). (**B**) Representative fluorescence images of the SIA component of the GCX stained with WGA-CF^TM^448A (red) in fixed cells using an inverted epi-fluorescence microscope. WGA mean cell intensity (MCI) was normalized to the respective number of cell nuclei stained with DAPI (blue). Data are expressed as mean ± S.E.M. (n = 3 independent cultures). *Scale bar=*20 μm*.* (**C**) Representative immunoblots and densitometric analyses of HO-1, NQO1 and GCLM expression in whole cell lysates presented relative to β-actin. Data denote mean ± S.E.M. (n = 5 independent cultures). *P < 0.05; **P < 0.01; ***P < 0.001 (2-way ANOVA). (For interpretation of the references to color in this figure legend, the reader is referred to the Web version of this article.)

To further investigate the role of SIA in shear stress mediated modulation of Nrf2 signaling, we silenced the endogenous sialidase NEU1. In EA. hy926 cells transduced with control shRNA, OSS enhanced NEU1 protein expression to a greater extent than USS, while flow conditioning of NEU1 knockdown cells did not alter sialidase levels compared to static culture (Fig. 4A). As NEU1 expression was previously associated with cell surface desialylation [48], we next assessed the effects of NEU1 silencing on SIA levels. NEU1 knockdown significantly enhanced SIA immunofluorescence in cells exposed to USS (Fig. 4B) and SIA levels were attenuated by disturbed compared to laminar flow. Moreover, enhanced SIA expression in the absence of NEU1 was associated with upregulation of Nrf2 target enzymes HO-1, NQO1 and GCLM in response to USS (Fig. 4C). Taken together, our findings suggest that SIA disruption reduces mechanosensitive activation of endogenous antioxidant systems by Nrf2 which is key for EC adaptation to oxidative stress in regions of high shear stress and may thus elicit dysfunctional EC phenotypes, investigated next.

### 3.5 Disturbed flow and SIA cleavage enhance mitochondrial ROS levels

**Fig. 5:**
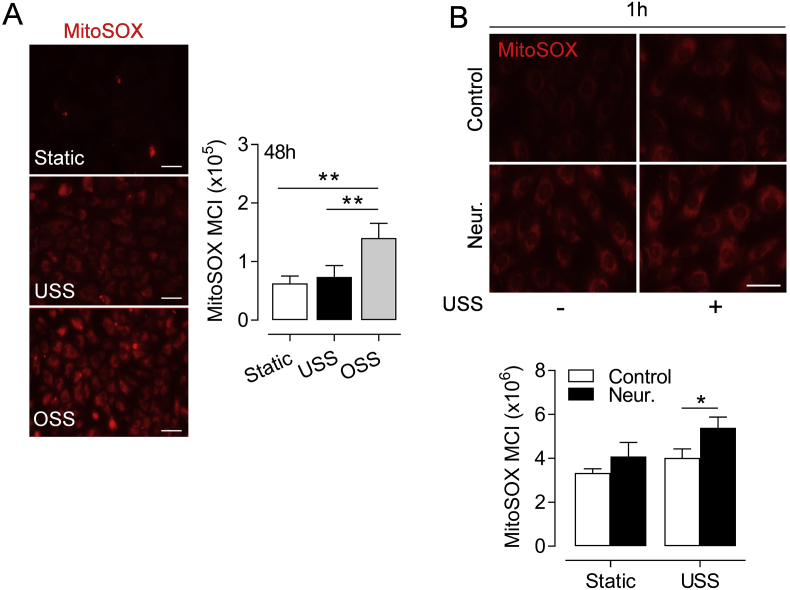
Effects of disturbed flow or SIA removal on mitochondrial ROS levels. (**A**) HUVEC were exposed to USS (15 dyn cm^-2^), OSS (±5 dyn cm^-2^, 1 Hz) or maintained in static conditions for 48 h. (**B**) HUVEC were treated with neuraminidase (Neur., 2 U ml^-1^, 30 min) before exposure to USS (15 dyn cm^-2^) for 1 h. At the end of all treatments, cells were incubated with the mitochondrial ROS indicator MitoSOX Red and maintained in static culture (30 min) before fixation and imaging. Cell nuclei were co-stained with DAPI which was omitted for clarity. Representative images and quantification of mean fluorescence intensity normalized to the number of cell nuclei are shown. Data denote mean ± S.E.M. (n = 4 different donors). *P < 0.05; **P < 0.01 (1 or 2-way ANOVA). *Scale bar=*50 μm. (For interpretation of the references to color in this figure legend, the reader is referred to the Web version of this article.)

Mitochondrial free radicals are important mechanosensitive secondary messengers responsible for inducible expression of Nrf2 targets in response to physiological flow [13,49]. However, mitochondrial ROS generation in response to disturbed flow is pro-apoptotic [50] and may contribute to EC-originated atherogenesis. Here, we observed enhanced levels of mitochondrial ROS in HUVEC exposed to prolonged OSS compared to laminar flow or with culture under static conditions (Fig. 5A). SIA removal with neuraminidase increased MitoSOX Red fluorescence in response to acute USS exposure (Fig. 5B) and a similar, albeit not statistically significant trend was observed in static cultures.

### 3.6 SIA removal promotes a pro-atherogenic EC phenotype in response to physiological flow

**Fig. 6:**
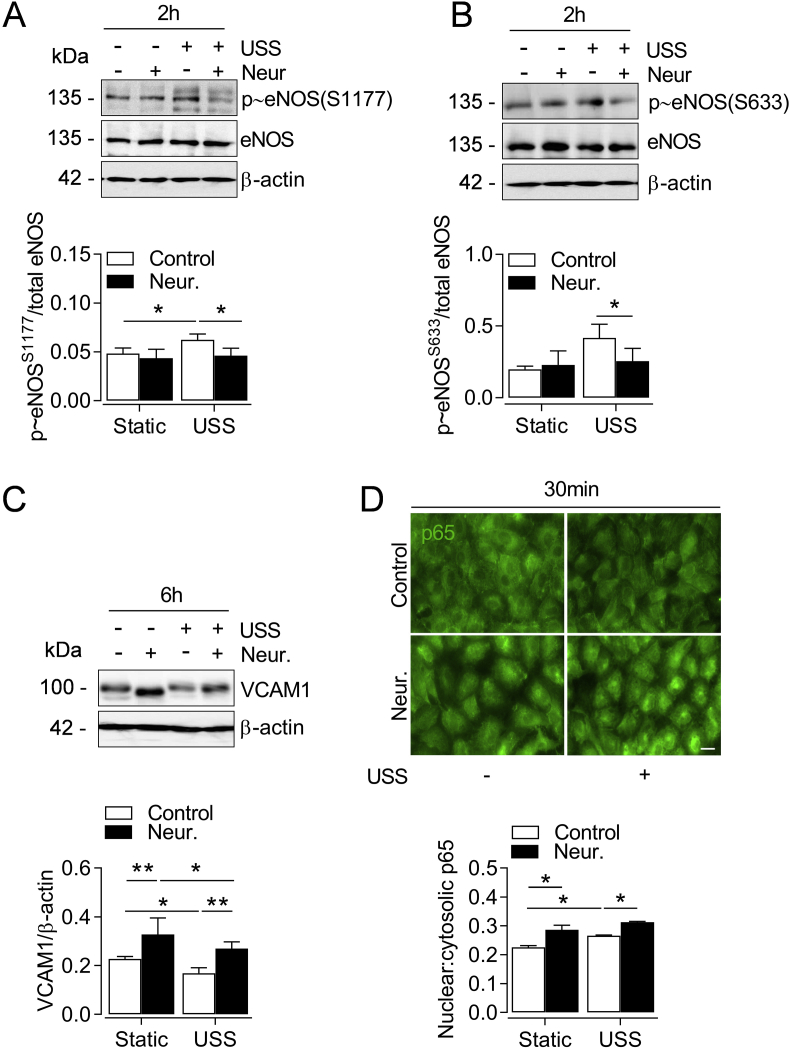
SIA cleavage attenuates eNOS phosphorylation by USS and induces VCAM-1. HUVEC were incubated with neuraminidase (Neur., 2 U ml^-1^, 30 min) before exposure to USS (15 dyn cm^-2^) for the indicated time points. (**A-B**) Representative immunoblots and densitometric analyses of eNOS phosphorylation at S1177 and S633 relative to β-actin and total eNOS levels. Data denote mean ± S.E.M. (n = 4–6 different donors). *P < 0.05 (2-way ANOVA). (**C**) Representative immunoblot and densitometric analysis of VCAM1 expression relative to β-actin. Data denote mean ± S.E.M. (n = 3 donors). *P < 0.05, **P < 0.01 (2-way ANOVA). (**D**) Representative images of p65 subunit cellular distribution (green) and quantification of nuclear-to-cytosolic fluorescence intensity in fixed cells. Cell nuclei were co-stained with DAPI which was omitted for clarity. Data denote mean ± S.E.M. (n = 4 different donors) of fluorescence intensity values from at least 300 cells per condition. *P < 0.05 (2-way ANOVA). *Scale bar=*20 μm. (For interpretation of the references to color in this figure legend, the reader is referred to the Web version of this article.)

Cleavage of SIA from the endothelial GCX also reduces flow-mediated NO bioavailability [51], therefore we next examined eNOS expression and phosphorylation following SIA removal. Neuraminidase did not affect total eNOS protein levels, however, in cells exposed to USS, SIA cleavage reduced phosphorylation of eNOS stimulatory sites Ser 1177 and 633 (Fig. 6A and B) which are critical for NO output [52,53]. Moreover, physiological EC function is disrupted in arterial regions susceptible to atherogenesis due to the diminished expression of transcription factors Klf2 and Klf4 [54,55]. The atheroprotective properties of Klf2 stimulation are partly mediated by induction of Nrf2 targets [16], therefore we assessed the effects of SIA disruption on shear-sensitive Klf2 and Klf4 regulation. Prolonged (24 h) EC exposure to OSS reduced Klf2 and Klf4 mRNA levels relative to USS (data not shown). Similarly, SIA cleavage with neuraminidase attenuated early induction of both Klf2 and Klf4 by USS which was restored following SIA re-growth at 24 h (Fig. S4).

Deficits in Klf2 and Klf4 expression promote atherosusceptible EC phenotypes partly via upregulation of vascular cell adhesion molecule 1 (VCAM-1) [55,56], and suppression of Nrf2 signaling by disturbed shear stress elicits vascular inflammation through similar mechanisms *in vivo* [9]. As SIA cleavage with neuraminidase attenuated USS-mediated Nrf2 responses and protein expression of Klf2 and Klf4, we next investigated whether it is also a pro-inflammatory EC stimulus. Prolonged (48 h) HUVEC exposure to physiological USS significantly reduced VCAM-1 protein levels compared to OSS and static culture (data not shown). Notably, SIA cleavage with neuraminidase enhanced VCAM-1 expression under both static and acute USS conditions (Fig. 6C)**.** Nuclear transcription factor-κB (NF-κB) is a key mediator of flow-sensitive VCAM-1 expression [57] and exhibits a biphasic pattern of initial activation and subsequent inhibition by prolonged laminar flow [58]. In agreement with these studies, acute cell exposure to USS increased nuclear translocation of the NF-κB p65 subunit, while SIA removal with neuraminidase further enhanced this effect (Fig. 6D) and promoted nuclear translocation of p65 under static conditions.

## 4 Discussion

This study is the first to demonstrate that the SIA component of the glycocalyx acts as a novel shear-sensitive regulator of endogenous Nrf2-mediated antioxidant defences in human EC. Moreover, we provide novel evidence that in the absence of SIA, EC cultured under USS have impaired eNOS phosphorylation, enhanced levels of mitochondrial ROS and the proinflammatory marker VCAM-1, thus phenotypically resembling EC exposed to pro-atherogenic OSS. Diminished SIA at arterial branch points exposed to disturbed flow [37] and, in combination with systemic stressors or risk factors such as age [24], predisposes these sites to atherogenesis. To recapitulate the endothelial desialylation that occurs in response to OSS, we removed SIA with exogenous neuraminidase, which has been shown to promote neointimal thickening and oxidized low density lipoprotein (ox LDL) accumulation *in vivo* [59], elicit pro-inflammatory responses [60] and enhance vascular permeability [61]. Given the wide range of EC homeostatic functions mediated by Nrf2 targeted transcription, here we describe a new, potentially anti-atherogenic role for SIA via Nrf2 signaling.

Using TEM [62], we demonstrated that USS enhanced the thickness of endothelial GCX *in vitro*, and although additional GCX components may contribute to this effect [63], our findings correlated with increased immunofluorescence of surface SIA and transcript levels of its biosynthetic enzyme GNE [40]. Studies in animal models have previously shown using TEM a reduced anatomical GCX depth at arterial regions exposed to disturbed blood flow patterns [64,65], which also exhibit reduced WGA staining [37,39]. This is consistent with our finding of reduced SIA immunofluorescence in cells exposed to OSS, possibly due to deficits in GNE and SLC35A1 transcription afforded by USS, but also via upregulation of CMAS, which generates cytosolic SIA-nucleotide donors [66]. The epimerase activity of GNE is tightly inhibited by cytosolic levels of the SIA-nucleotide pairs which are normally concentrated into the *trans*-Golgi by the SLC35A1 antiporter [42]. Enhanced CMAS transcripts and reduced SLC35A1 expression in EC exposed to OSS may therefore rise the cytosolic SIA-nucleotide donor concentration and further impede GNE function. Upregulation of DNA-methyltransferases by disturbed flow [67] may also reduce GNE transcription via promoter CpG islet hypermethylation [68]. Notably, tissue hyposialylation due to deficits in GNE activity leads to age-related neuronal loss [69] and enhances oxidative stress in the skeletal muscle of patients with GNE myopathy [70]. Maintenance of surface SIA by shear-sensitive regulation of its biosynthesis and degradation is therefore crucial for the regulation of vascular homeostasis.

Moreover, the enhanced levels of free SIA we measured in the USS-conditioned culture medium suggested increased SIA biosynthesis and turnover as shown previously for the GCX component HS [45]. Serum total SIA is elevated following acute myocardial infarction and in patients with type II diabetes and correlates with the severity of atherosclerosis [71] and circulating markers of oxidative stress [72]. Notably, SIA is susceptible to oxidative cleavage by ROS [73] and oxidative desialylation of plasma proteins such as LDL also contributes to the circulating SIA pool [74]. It is possible that enhanced generation or diminished scavenging of ROS observed in response to OSS further contributed to reduced expression of SIA. Taken together, our results suggest a multifaceted dysregulation of biosynthesis and surface retention of SIA in response to OSS.

The decline in cellular SIA in EC exposed to oscillatory flow was also associated with enhanced expression of NEU1. The endogenous sialidase NEU1 resides in two subcellular compartments with distinct homeostatic functions; lysosomal NEU1 regulates cytosolic SIA levels by recycling sialoconjugates [75], whereas in the plasma membrane it initiates inflammatory cascades via desialylation of surface molecules such as ICAM-1 [76] and TLR4 [77]. NEU1-mediated SIA cleavage also inhibits Akt signaling downstream of integrin α5β1 [78] and the latter mediates proinflammatory NF-kB activation in response to OSS [79]. In the present study, NEU1 silencing enhanced SIA expression in cells exposed to USS but had little effect on SIA levels following exposure to OSS, likely due to the sustained deficit in SIA biosynthesis described above. Importantly, enhanced SIA expression as a result of NEU1 knockdown upregulated the expression of Nrf2 target antioxidant enzymes in response to USS. Pharmacologic or genetic inhibition of NEU1 activity has been shown to reduce serum cholesterol and alleviate vascular dysfunction that underlies atherogenesis in the ApoE−/− mouse [48,80]. This suggests that shear-sensitive NEU1 expression may determine vascular sites of atherogenesis, and based on our findings, we propose that this is partly mediated via Nrf2-dependent regulation of endothelial redox signaling.

As reported previously [10], USS enhanced the expression of endogenous antioxidant defenses via Nrf2 signaling but only in the presence of intact SIA. Functional nuclear accumulation of Nrf2 in response to USS is mediated by PI3K and downstream Akt and protein kinase C activity [7,14], thus reduced Akt activation following SIA removal possibly attenuated Nrf2 responses to USS. Akt signaling also represses GSK3β activity [47], which is known to promote Nrf2 nuclear export [81] and its Keap-1 independent cytosolic degradation [82]. This may contribute to the diminished induction of Nrf2 nuclear accumulation by USS following SIA cleavage. Moreover, direct tethering of the Keap-1/Nrf2 complex on the outer mitochondrial membrane is postulated to maintain mitochondrial redox homeostasis [83], thus impaired Nrf2 activation in the absence of SIA has likely enhanced mitochondrial ROS accumulation in response to USS. In agreement with our findings, vascular ROS accumulation is observed in porcine femoral arteries perfused with neuraminidase [26] and extracellular ROS levels are upregulated when EC are exposed to neuraminidase in the presence of a phagocytic stimulus [84]. Based on evidence that SIA can directly interact with H_2_O_2_ and •OH [85,86], it is possible that the antioxidant defences conferred by SIA-mediated scavenging were diminished after removal with neuraminidase. Although neuraminidase does not disrupt the extracellular superoxide dismutase (ecSOD) [26] which is bound to the HS component of the GCX [87], in the latter study, ecSOD did not provide sufficient antioxidant defence in the absence of SIA. Our finding of diminished Nrf2-mediated antioxidant responses therefore represents a novel mechanistic link between SIA disruption and vascular redox imbalance.

Enhanced oxidative stress as a result of endothelial desialylation with neuraminidase has profound implications for vascular tone regulation as it contributes to impaired NO-mediated vasodilation [26]. Although the pro-oxidant environment directly decreases NO bioavailability [88], SIA removal with neuraminidase also inhibits flow-mediated NO production [51] and reduces soluble guanylate cyclase activity *ex vivo* [25]. Our finding of reduced eNOS phosphorylation in EC treated with neuraminidase therefore implicates SIA in mechanotransduction of USS for NO-mediated vasomotor control. This is in line with previous reports of reduced eNOS Ser^1177^ [89] and Ser^633^ [90] phosphorylation following disruption of other key GCX components. In this study, neuraminidase did not affect the expression of the major GCX glycosaminoglycan HS, however removal of SIA can decrease the negative surface charge that, in turn, distorts GCX structure and possibly alters mechanical force transmission for intracellular signaling throughout the GCX layer [20].

We also demonstrated for the first time that SIA cleavage with neuraminidase upregulates endothelial VCAM-1 expression. As attenuated Nrf2 signaling has been directly implicated in the pathogenesis of vascular inflammation, SIA disruption may additionally initiate proinflammatory events via redox dysregulation. SIA deterioration due to systemic inflammation [91] or cleavage by neuraminidase [60] promotes inflammatory cell trafficking on healthy vessels and desialylation of VCAM-1 enhances EC adhesiveness under laminar flow [92]. Additionally, Nrf2 knockout mice exhibit enhanced VCAM-1 expression in normally atheroprotected aortic regions [9] and VCAM-1 levels are enhanced by Nrf2 silencing in EC exposed to shear stress *in vitro* [93]. In the latter study this is partly alleviated by antioxidant treatment, therefore enhanced mitochondrial ROS observed in our study in the absence of SIA is likely to have contributed to NF-kB activation [94]. Enhanced VCAM-1 expression has been observed following endothelial Klf4 depletion [55] whereas overexpression of Klf2 and Klf4 attenuates NF-kB assembly and VCAM-1 promoter activation [55,56]. Therefore, the interactions between SIA and the mechanosensitive transcription factors Klf2 and Klf4 are likely to protect against proinflammatory changes in arterial regions exposed to USS.

Perturbations in GCX underly EC dysfunction in vascular pathologies associated with oxidative stress such as diabetes, stroke, hypertension and atherosclerosis [95]. Moreover, the age-related decline in adaptive cellular responses to oxidative stress, especially blunting of vascular Nrf2 antioxidant signaling, plays a key role in the accumulation of oxidative modifications that contribute to macromolecular damage and inflammation in CVD [96]. Microvascular dysfunction has also been linked to age-related GCX decline [97], therefore therapeutic strategies that mitigate GCX deterioration are likely to reduce the risk and severity of CVD in ageing [98]. Notably, experimental restoration of SIA has been shown to be efficacious against atherosclerosis [99], obesity-related hypertension [100] and age-related renal microvascular dysfunction [101]. Furthermore, it was recently reported that GCX enhancement by the GAG supplement sulodexide activates Nrf2 signaling to confer cytoprotection against ischaemia-reperfusion injury [102]. Further studies are thus warranted to further elucidate interactions between GCX components in coordinating Nrf2-regulated antioxidant defences. In summary, our findings provide a novel insight into the molecular mechanisms by which the endothelial GCX maintains Nrf2-mediated redox homeostasis and highlights the therapeutic potential of targeting SIA metabolism to ameliorate vascular dysfunction in atherogenesis and age-related CVD.

## Authors contributions

R.C.S. and M.F. conceptualized the study and were awarded the grant funding; P-M. P. developed the methodology and performed the experiments; P.K. assisted with the collection of umbilical cords and performed some of the experiments; G.V. and R.F. assisted with the TEM analyses of the glycocalyx; P-M.P., R.C.S., S.J.C. and G.E.M drafted the manuscript which was reviewed by all authors. R.C.S. is the guarantor of this study, with responsibility for the integrity of the data and accuracy of the data analysis.

## Declaration of competing interest

Authors declare no conflicts of interest.
