# Supplementary material for: Glycocalyx sialic acids regulate Nrf2-mediated signaling by fluid shear stress in human endothelial cells
Source: Redox Biol. 2020 Nov 28;38:101816. doi: 10.1016/j.redox.2020.101816 (PMC7750408; doi:10.1016/j.redox.2020.101816)
Supplement: Multimedia component 1 [file mmc1.docx]

**Supplementary Data**


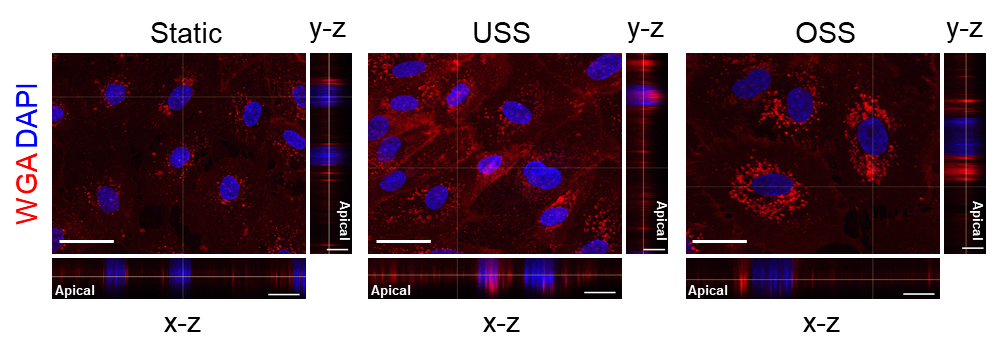


**Fig. S1. Differential distribution of SIA in response to laminar and disturbed shear stress.** HUVEC were exposed to USS (15 dyn cm^-2^), OSS (±5 dyn cm^-2^, 1 Hz) or maintained in static conditions for 48 h. Representative confocal images of the SIA component of the GCX stained with WGA-CF^TM^448A (red) and the cell nuclei stained with DAPI (blue) in fixed cells. The y- and x- volume reconstructions of the z-plane (20μm depth, inter-slice distance, 0.1μm) are shown around the main x-y panel for each condition. Images are representative of n=3 different donors. *Scale bars: x-y=20μm, x-z=10μm, y-z=5μm.*


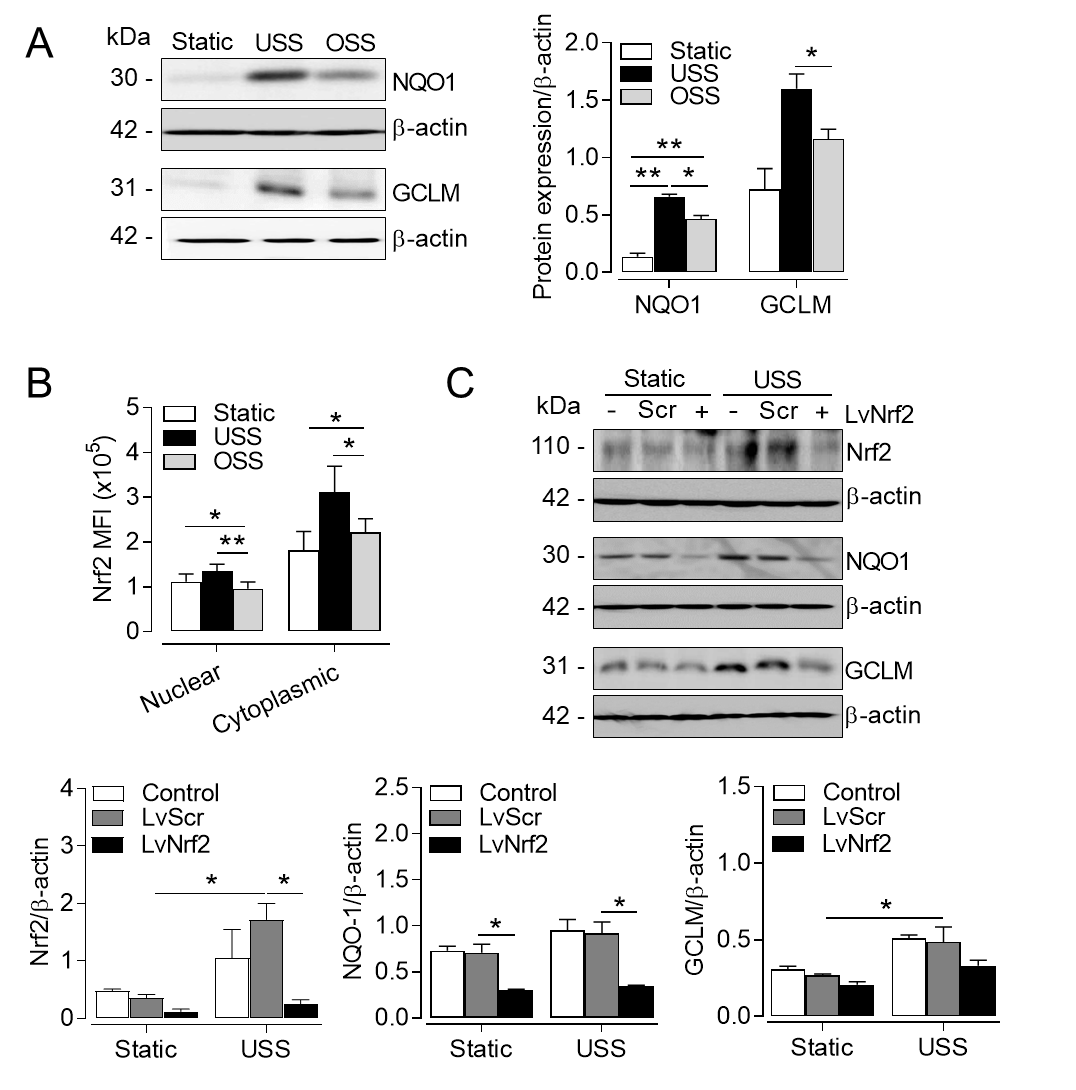


**Fig. S2. Laminar shear enhances Nrf2 nuclear translocation and antioxidant enzyme expression *in vitro***. HUVEC were exposed to USS (15 dyn cm^-2^), OSS (±5 dyn cm^-2^, 1 Hz) or maintained in static conditions for 48 h. (**A**) Representative immunoblots and densitometric analyses of NQO1 and GCLM expression is shown relative to β-actin. Data denote mean ± S.E.M. (n=4-6 donors). *P<0.05; **P<0.01 (1-way ANOVA). (**B**) Quantification of nuclear and cytoplasmic fluorescence intensity of Nrf2 in fixed cells. Data from at least 50 cells per condition are presented as mean ± S.E.M. (n=3 donors). *P<0.05; **P<0.01 (1-way ANOVA). (**C**) EA.hy926 cells were either transduced with lentiviral particles containing Nrf2 silencing shRNA (LvNrf2) or scrambled sequences (Scr) or left untransfected (Control). Whole cell Nrf2, NQO1 and GCLM protein expression relative to β-actin following application of USS (15 dyn cm^-2^) for 24 h. Data denote mean ± S.E.M. (n=3 independent experiments). *P<0.05 (2-way ANOVA).

**
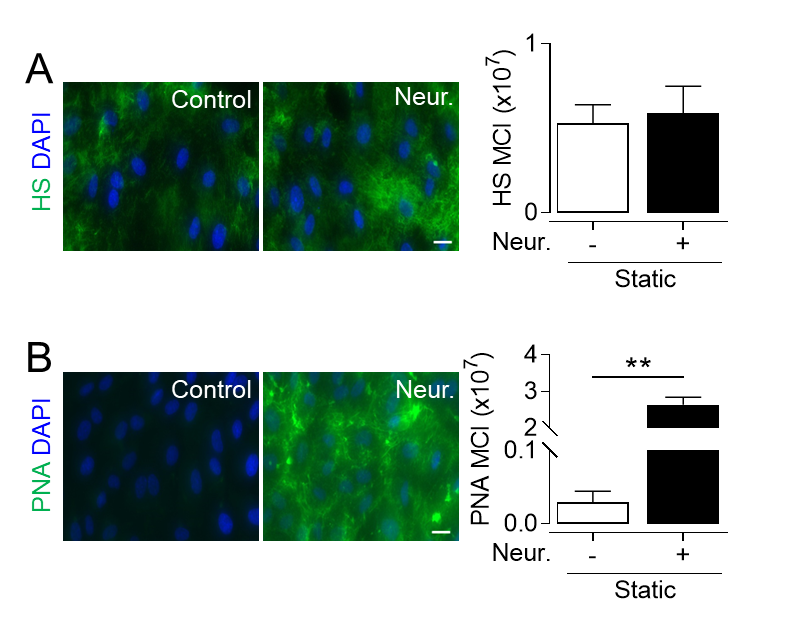
**

**Fig. S3. Neuraminidase selectively removes the SIA component of the GCX**. HUVEC maintained in static culture were incubated with neuraminidase (Neur., 2 U ml^-1^, 30 min) and fixed cells were stained with (**A**) the anti-N-sulphated HS 10E4 epitope followed by an Alexa Fluor 568 secondary antibody (green) or (**B**) peanut lectin (PNA-CFTM568A) from Arachis hypogaea (green) that binds to subterminal β-galactose residues. Representative images of HS, PNA and cell nuclei stained with DAPI (blue). Data are expressed as mean cell intensity (MCI) normalized to the respective number of cell nuclei per FOV and represent mean ± S.E.M. (n=4 donors) of at least 50 cells per condition. **P<0.01 (Student’s t-test). *Scale bar=20μm.*


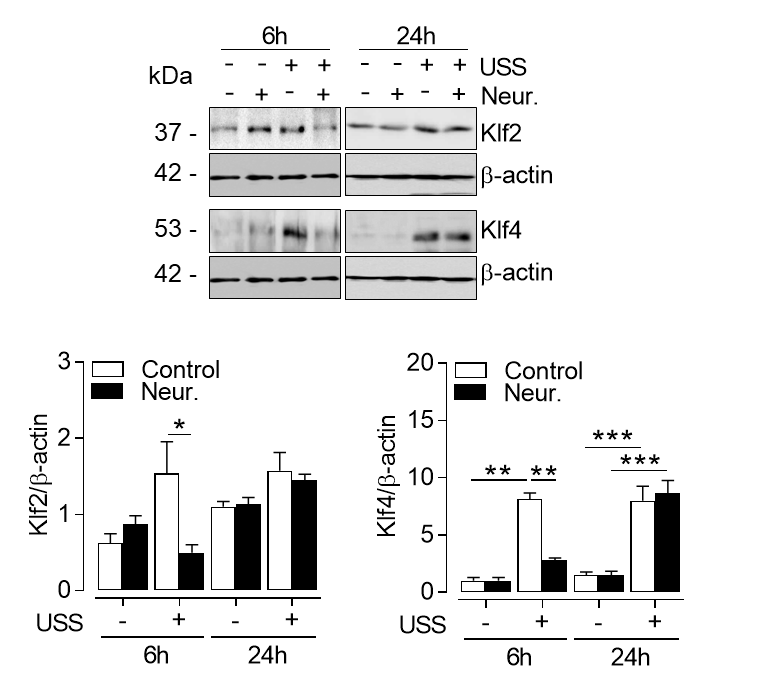


**Fig. S4.** **SIA cleavage attenuates the induction of Klf2 and Klf4 by USS.** HUVEC were incubated with neuraminidase (Neur., 2 U ml^-1^, 30 min) before exposure to USS (15 dyn cm^-2^) for the indicated time points. Representative immunoblots and densitometric analyses of Klf2 and Klf4 protein expression relative to β-actin. Data denote mean ± S.E.M. (n=4-6 different donors). *P<0.05 (2-way ANOVA).
